# 

**Published:** 1862-03

**Authors:** 


New Series, Vol. 5, No. 3. Whole Series, Vol. 19, No. 3.

### THE CHICAGO MEDICAL JOURNAL.

DANIEL BRAINARD, M. D.,
J. ADAMS ALLEN, M. D.,
Editors and Proprietors.

MARCH, 1862.

CHICAGO:
WM. PIGOTT, BOOK AND JOB PRINTER, 130 CLARK STREET.
1862.

